# Interrogating the architecture of cancer genomes

**DOI:** 10.1186/gb-2011-12-s1-i4

**Published:** 2011-09-19

**Authors:** Peter Campbell

**Affiliations:** 1 Cancer Genome Project, Wellcome Trust Sanger Institute, Hinxton, UK

## 

Cancer is driven by mutation. Using massively parallel sequencing technology, we can now sequence the entire genome of cancer samples, allowing the generation of comprehensive catalogs of somatic mutations of all classes. Bespoke algorithms have been developed to identify somatically acquired point mutations, copy number changes and genomic rearrangements, which require extensive validation by confirmatory testing. The findings from our first handful of genomes illustrate the potential for next-generation sequencing to provide unprecedented insight into mutational processes, cellular repair pathways and gene networks associated with cancer development. I will also review the possible applications of these technologies in a diagnostic and clinical setting and the potential routes for translation.

